# Low-dose Fosphenytoin for Aborting Acute Trigeminal Neuralgia Pain: A Case Report

**DOI:** 10.5811/cpcem.1603

**Published:** 2023-08-09

**Authors:** Jamie Baydoun, Alexander Lin, Jared Miya

**Affiliations:** * University of Nevada Las Vegas School of Medicine, Department of Emergency Medicine, Las Vegas, Nevada; † University Medical Center of Southern Nevada, Department of Psychiatry and Behavioral Health, Las Vegas, Nevada; ‡ Sound Emergency Physicians, Department of Emergency Medicine, Las Vegas, Nevada

**Keywords:** fosphenytoin, trigeminal neuralgia, emergency department management, case report

## Abstract

**Introduction:**

While the typical treatment for trigeminal neuralgia is carbamazepine, the dose must be gradually titrated over time to achieve pain control, which makes the drug a less than ideal candidate for treatment for acute exacerbation of pain due to trigeminal neuralgia in the emergency department (ED) setting. The literature for other effective treatments for acute exacerbations of trigeminal neuralgia is currently lacking. We discuss a case where intravenous (IV) fosphenytoin was used for treatment of acute pain due to trigeminal neuralgia in the ED.

**Case Report:**

This is a case of a 35-year-old male diagnosed with trigeminal neuralgia who presented with acute facial pain. His history and physical exam were consistent with an acute exacerbation of his trigeminal neuralgia. The patient was refractory to multiple doses of standard pain medication in the ED, and the decision was made to attempt IV fosphenytoin to relieve his pain. He was given 250 milligrams of fosphenytoin that was infused via IV over 10 minutes. By the end of the infusion, the patient had reported complete resolution of his pain.

**Conclusion:**

Fosphenytoin is a viable treatment option for pain relief in patients with acute exacerbation of trigeminal neuralgia. It may be a more favorable drug to use in the ED for acute pain given that carbamazepine must be titrated to effect. It is also possible that lower doses of fosphenytoin may provide equally beneficial analgesic effect than what is described in the literature, as pain relief was achieved in our case with approximately 3 milligrams/kilogram of fosphenytoin.

## INTRODUCTION

Trigeminal neuralgia (TN) is a rare condition with severe, debilitating symptoms that is best controlled quickly. Trigeminal neuralgia is divided into three classifications: primary, also referred to as classic, secondary, and idiopathic. Primary TN is thought to be caused by vascular compression of the nerve root at the pons, and secondary TN is caused by demyelinating diseases or mass effect and compression. The most effective medication described in the literature for maintenance of primary TN is carbamazepine.^1^–^2^ Other potential treatments include surgical decompression or local peripheral nerve block. Unfortunately, carbamazepine often needs to be titrated to alleviation of clinical symptoms over days to weeks, and this makes it a poor emergency department (ED) choice for abortion of acute TN flares.

## CASE REPORT

We report a case of a 35-year-old male diagnosed with trigeminal neuralgia eight months prior who presented with two days of intractable 10/10 left facial pain with radiation from his jaw to his temple. This pain was exacerbated by clenching of his jaw and chewing. He described his pain as sharp and shooting, lasting only seconds, and consistently self-resolving. Over the two days prior to his ED visit, he had more frequent episodes of pain, and endorsed up to 30 episodes a day. His pain was refractory to multiple over-the-counter pain medications. The day of presentation he had six hours of near-constant clusters of severe shooting pain. His physical exam revealed normal vital signs, normal head, eyes, ears, nose, and throat exam except for hyperalgesia of his left face. Cranial nerves 2–12 were intact. He had normal speech, symmetric motor and sensation to all four extremities, and a normal gait. He had no meningismus upon examination of his neck.

In our ED, he was initially treated with a cocktail of ibuprofen, diphenhydramine, prochlorperazine, and one liter of normal saline. The patient had no resolution of symptoms 45 minutes after administration of this “migrane cocktail.” The history and physical exam were consistent with a trigeminal neuralgia crisis. To treat the patient’s neuropathic pain, he was given 250 milligrams (mg) of fosphenytoin that was infused intravenously (IV) over 10 minutes. At the end of the infusion, his pain had completely resolved. He was discharged with a neurology follow-up and a prescription for carbamazepine. Chart review six months after the patient was discharged did not show any other ED visits listed after discharge.

## DISCUSSION

Typical treatment for trigeminal neuralgia includes carbamazepine; however, the dose must be gradually increased to achieve pain control. There is currently a paucity of evidence regarding effective alternative treatments for acute exacerbations of TN. The American Academy of Neurology does not recommend, or refute, the use of any medication for acute TN.^1^ The European Academy of Neurology suggests that opioids are ineffective for management of acute TN.^2^

The European Academy of Neurology guidelines do state that in acute exacerbations of TN IV infusions of fosphenytoin or lidocaine may be used as treatment methods. Furthermore, a systematic review by Moore et al. showed limited data suggesting that lidocaine, sumatriptan, phenytoin, or fosphenytoin could be effective rescue analgesic strategies in acute exacerbations of primary TN.^3^ In a placebo-controlled crossover trial of 20 patients the administration of IV infusion of lidocaine at 5 mg/kilogram (kg) over one hour showed some relief in pain for up to 24 hours after infusion.^4^ However, IV lidocaine administration requires continuous cardiac monitoring with electrocardiogram and frequent blood pressure checks. Another small, placebo-controlled crossover trial demonstrated that 3 mg subcutaneous injection of sumatriptan could provide improvement in pain relief. However, the mean duration of pain relief was only about eight hours in that study.^5^

**CPC-EM Capsule**

What do we already know about this clinical entity?

*The most effective medication for maintenance of trigeminal neuralgia is carbamazepine however, it often needs to be titrated over many days, making it a poor choice for aborting acute flares.*

What makes this presentation of disease reportable?

*Literature for other effective treatments for acute trigeminal neuralgia flare is lacking. We discuss a case where fosphenytoin was used successfully for acute trigeminal neuralgia flares.*

What is the major learning point?

*Fosphenytoin is a viable treatment option for pain relief in patients presenting to the emergency department (ED) with acute exacerbation of trigeminal neuralgia.*

How might this improve emergency medicine practice?

*Fosphenytoin can be an effective option for aborting pain in acute trigeminal neuralgia pain due to its rapid onset of relief, low cost and wide availability in the ED.*

Fosphenytoin is a phosphate ester prodrug that is directly metabolized by plasma esterases to the active moiety phenytoin. Phenytoin is approved by the US Food and Drug Administration for seizures; however, off-label phenytoin use for neuropathic pain has been described in the literature.^6^ Phenytoin is known to stabilize neuronal membranes by decreasing the influx of sodium ions, which in turn decreases the generation of nerve impulses.^7^ Sodium channel blockade is the most likely mechanism of action by which phenytoin exerts its analgesic effect in trigeminal neuralgia.^8^

There are case reports and retrospective reviews demonstrating that fosphenytoin achieved significant pain control in patients with acute neuropathic and TN pain. McCleane was one of the first investigators to report the effectiveness of IV phenytoin as a treatment for neuropathic pain. In this double-blinded, placebo crossover study, patients with neuropathic pain were give either a placebo or 15 mg/kg of phenytoin. There was a significant reduction in burning pain (*P*<0.05), shooting pain (*P*<0.001), sensitivity (*P*<0.001), and overall pain (P<0.005).^9^ While there were no cases of TN crisis in McCleane’s study, there have since been reports showing the effectiveness of IV fosphenytoin in acute TN pain crisis. Cheshire reported using IV fosphenytoin in three cases of acute TN with complete resolution of pain for two days, which allows for titration of oral medications.^10^ Another case report by Vargas and Thomas demonstrated an improvement of pain down to 2/10 after a 15 mg/kg dose of IV fosphenytoin.^11^ A retrospective study performed by Schnell et al. in Argentina reviewed 73 IV fosphenytoin infusions for TN crisis. In this review 85% of cases had immediate relief, with 26% of patients experiencing adverse effects, with dizziness and nausea as the most common.^12^

Our patient received a much lower dose of fosphenytoin, roughly 3 mg/kg (250 mg total), than what has been described in the literature. We based this dose off recommendations in the tertiary drug reference Lexicomp, which lists fosphenytoin as an off-label use for rescue therapy for trigeminal neuralgia. The dose range listed was IV 250 mg–1gram as a one-time dose or 15 mg/kg. We decided to opt for the lower end of dosing to potentially minimize adverse effects. In the Cheshire case series, interval dosing of fosphenytoin was used, starting at 100 mg with reassessment every 10 minutes for additional dosing.^10^ Our plan was to use the lowest dose (250 mg) based on our drug reference in Lexicomp and then increase by 100 mg to a total of 1 gram as needed based on reassessment of symptoms every 10 minutes. Since our patient had resolution of symptoms and continued to be pain-free on multiple reassessments, we did not need to give additional doses. Our patient had complete resolution with just one 3 mg/kg dose of fosphenytoin, which suggests the analgesic effect of fosphenytoin in trigeminal neuralgia may be achieved with more conservative dosing. This is important to note given that hydantoins are known to have dose-dependent, life-threatening toxicities such as coma, seizures, hypotension, and bradyarrhythmias.

## CONCLUSION

In our case, the patient had complete alleviation of symptoms immediately after infusion of fosphenytoin. We believe fosphenytoin is an excellent treatment option for acute trigeminal neuralgia because it is available in most EDs, is reasonably priced, and has been shown in case studies and retrospective reviews to have rapid onset of relief for acute TN. We were able to use a lower dose of fosphenytoin to achieve pain relief in our patient’s acute TN crisis. Randomized controlled trials should be performed to determine if and at what dose fosphenytoin is efficacious for treatment of acute trigeminal neuralgia pain crisis.
